# Long-term survival after hepatectomy for metachronous liver metastasis of pancreatic ductal adenocarcinoma: a case report

**DOI:** 10.1186/s40792-020-00924-8

**Published:** 2020-07-03

**Authors:** Chikanori Tsutsumi, Toshiya Abe, Tomohiko Shinkawa, Kazuyoshi Nishihara, Sadafumi Tamiya, Toru Nakano

**Affiliations:** 1 Department of Surgery, Kitakyushu Municipal Medical Center, 2-1-1 Bashaku, Kokurakita-Ku, Kitakyushu, 802-0077 Japan; 2 Department of Pathology, Kitakyushu Municipal Medical Center, Kitakyushu, Japan

**Keywords:** Hepatectomy, Long-term survival, Liver metastasis, Pancreatic ductal adenocarcinoma

## Abstract

**Background:**

Pancreatic ductal adenocarcinoma (PDAC) is among the most aggressive malignancies. The prognosis for recurrence after surgery is extremely unfavorable, and liver metastasis of PDAC confers poor prognosis despite resection.

**Case presentation:**

A 51-year-old man was admitted to our hospital for further examination and treatment, including surgery for a pancreatic tumor. On close inspection, he was suspected to have pancreatic head cancer without enlarged lymph nodes or distant metastasis, and pancreatoduodenectomy with D2 lymph node dissection was performed. A postoperative pathological examination revealed well-differentiated invasive ductal adenocarcinoma with lymph node metastasis (stage IIB; pT2N1M0). Postoperatively, he received adjuvant chemotherapy containing gemcitabine for 1 year. Eight years after the radical surgery, his serum carbohydrate antigen 19-9 level was elevated, and computed tomography (CT) and magnetic resonance imaging revealed a well-circumscribed 10-mm mass in liver segment 5. Positron emission tomography/CT also revealed high fluorine-18-fluorodeoxyglucose uptake only in this hepatic tumor. Accordingly, the patient was diagnosed with a solitary liver metastasis of PDAC. As the liver metastasis was isolated and identified long after the initial surgery, we decided to resect it using laparoscopic partial hepatectomy of segment 5. Histopathological examination confirmed liver metastasis of PDAC and the patient received adjuvant chemotherapy containing S-1. No evidence of recurrence has been seen for 11 years since the pancreatoduodenectomy and 3 years since the hepatic resection.

**Conclusions:**

Cases of metachronous liver metastasis of PDAC after radical surgery, in which patients exhibit long-term survival without recurrence after hepatectomy, are extremely rare. Hepatectomy may confer long-term survival, and the time to postoperative recurrence and the number of liver metastases may be useful criteria for deciding whether to perform hepatic resection.

## Background

Pancreatic ductal adenocarcinoma (PDAC) is among the most aggressive malignancies. Despite recent progress in diagnostic imaging modalities, surgical procedures, and chemotherapy, its prognosis remains poor [1, 2]. Here, we report an extremely rare case of metachronous liver metastasis of PDAC after pancreatoduodenectomy (PD) in a patient who survived without a recurrence for 11 years after PD and 3 years after hepatectomy.

## Case presentation

**Fig. 1:**
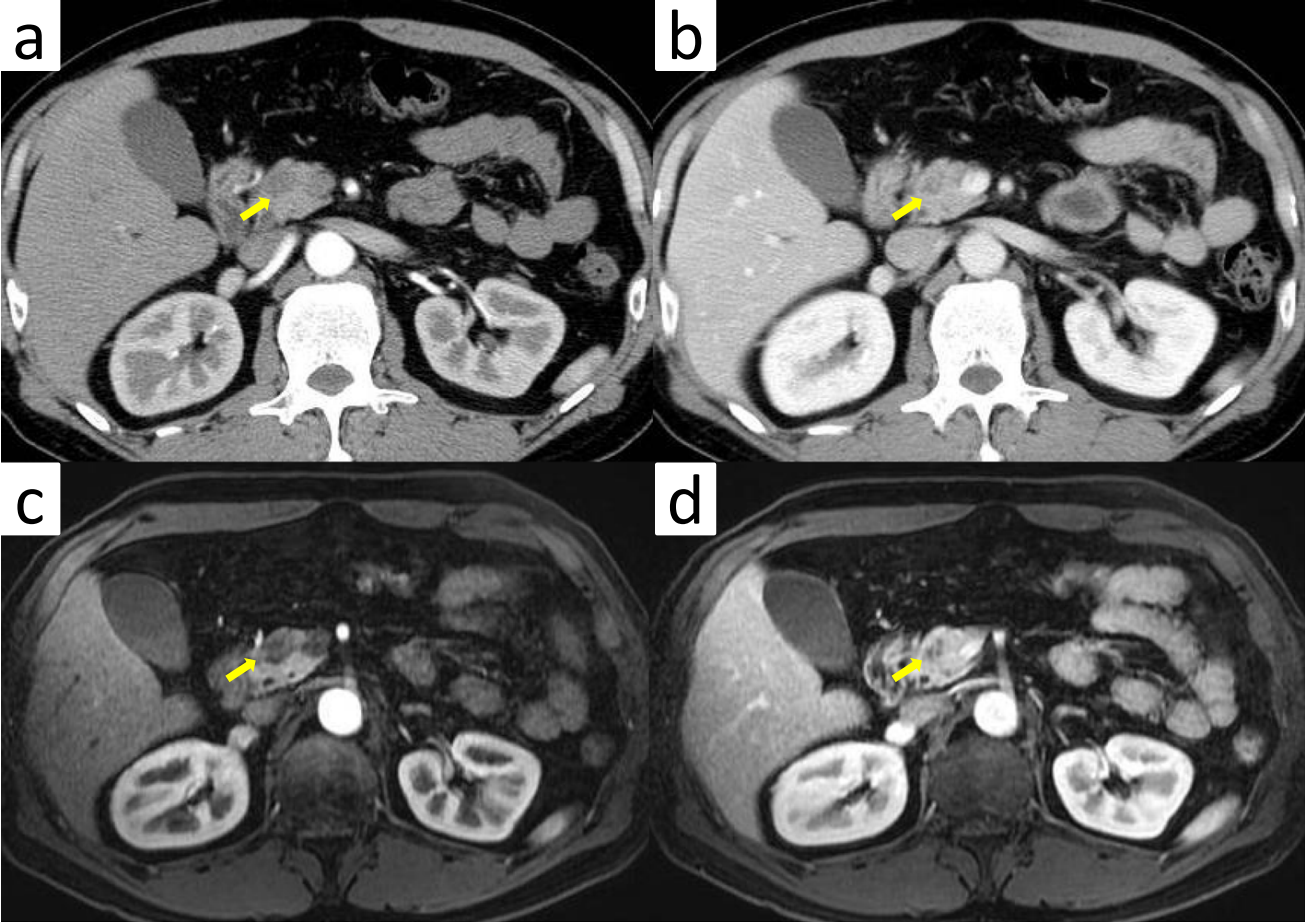
Contrast-enhanced CT and Gd-EOB-DPTA–enhanced MRI revealed a nodule that was gradually contrasted in the pancreatic head (arrow). **a** Arterial phase of CT. **b** Portal phase of CT. **c** Early phase of MRI. **d** Late phase of MRI. CT, computed tomography; Gd-EOB-DPTA, gadolinium-ethoxybenzyl-diethylenetriamine; MRI, magnetic resonance imaging

**Fig. 2:**
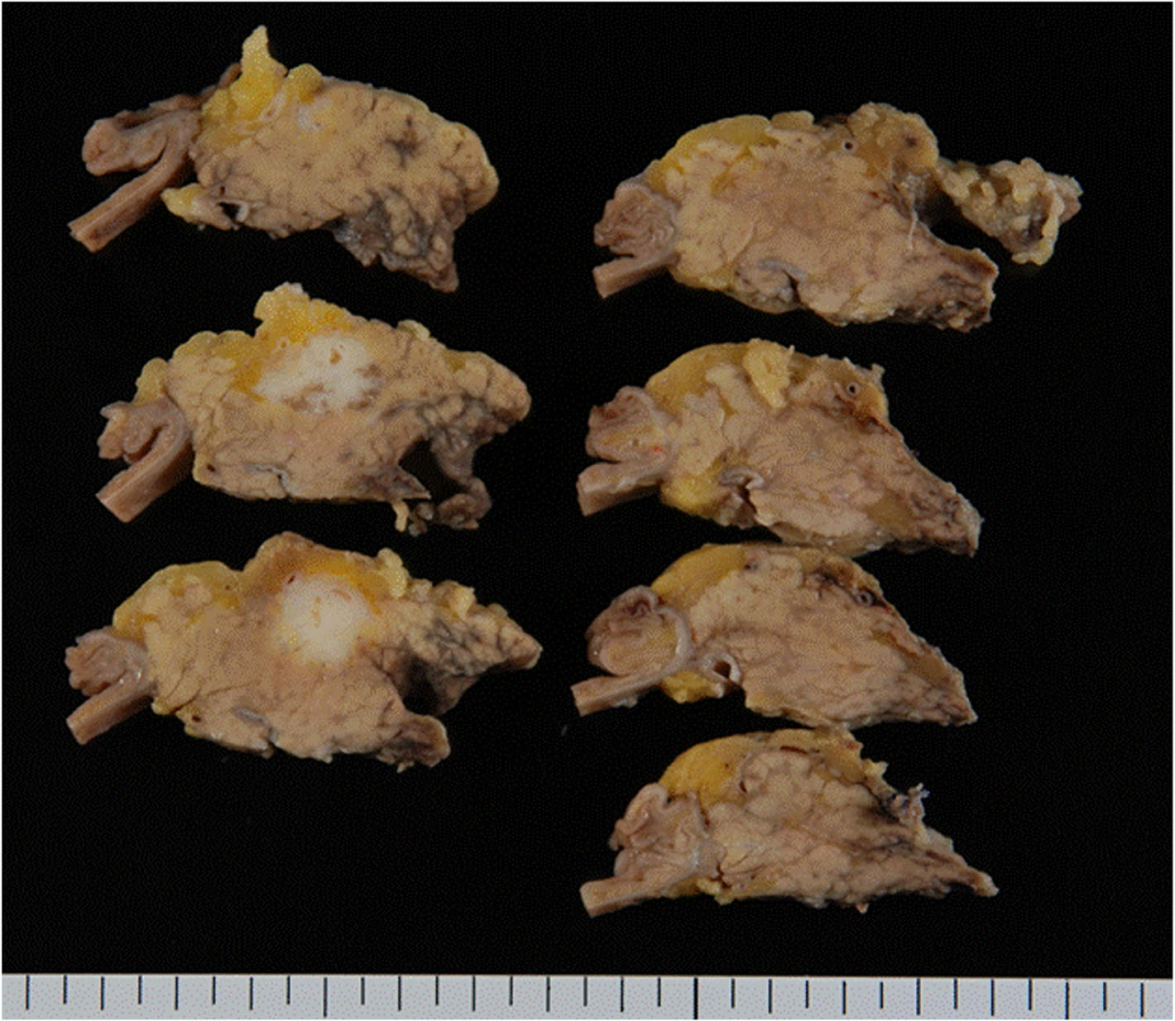
Macroscopic examination of the resected specimen identified a well-circumscribed 21 × 15mm nodule in the pancreatic head (arrow)

**Fig. 3:**
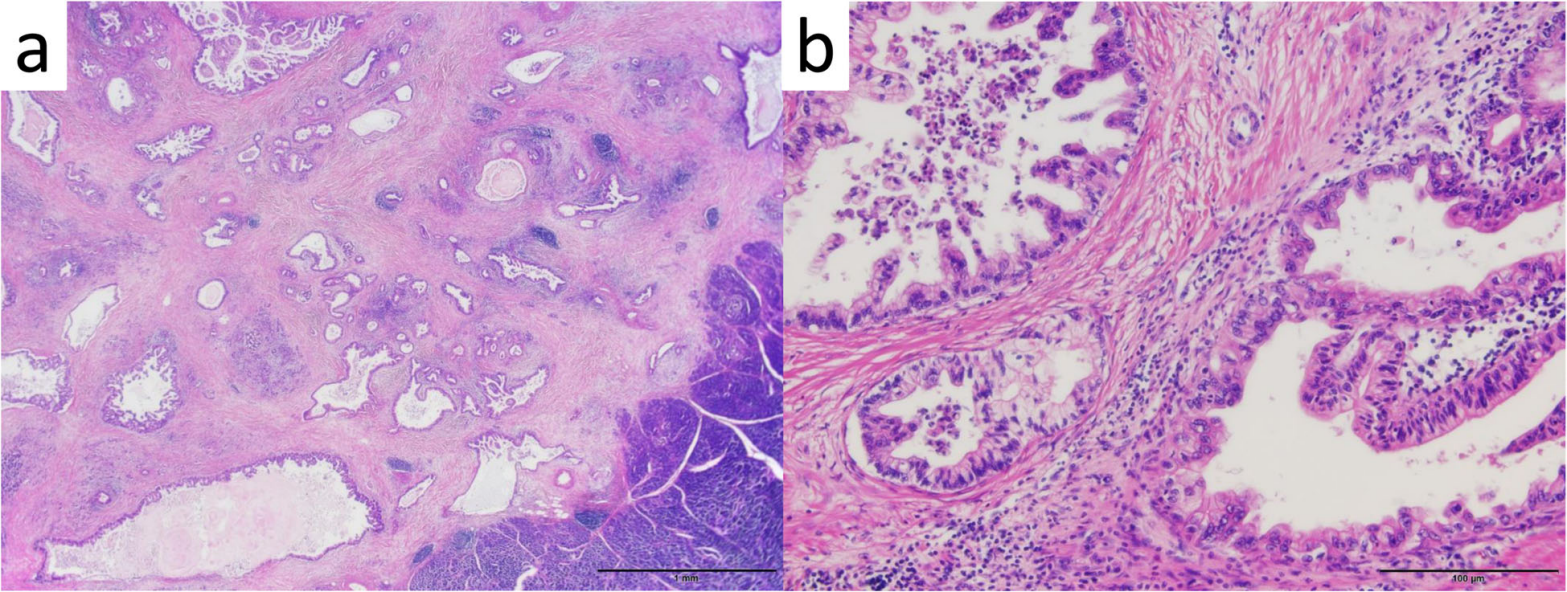
Histopathological findings (hematoxylin–eosin staining) showing well-differentiated invasive ductal adenocarcinoma with lymphatic invasion. **a** × 4 and **b** × 40 original magnification

**Fig. 4:**
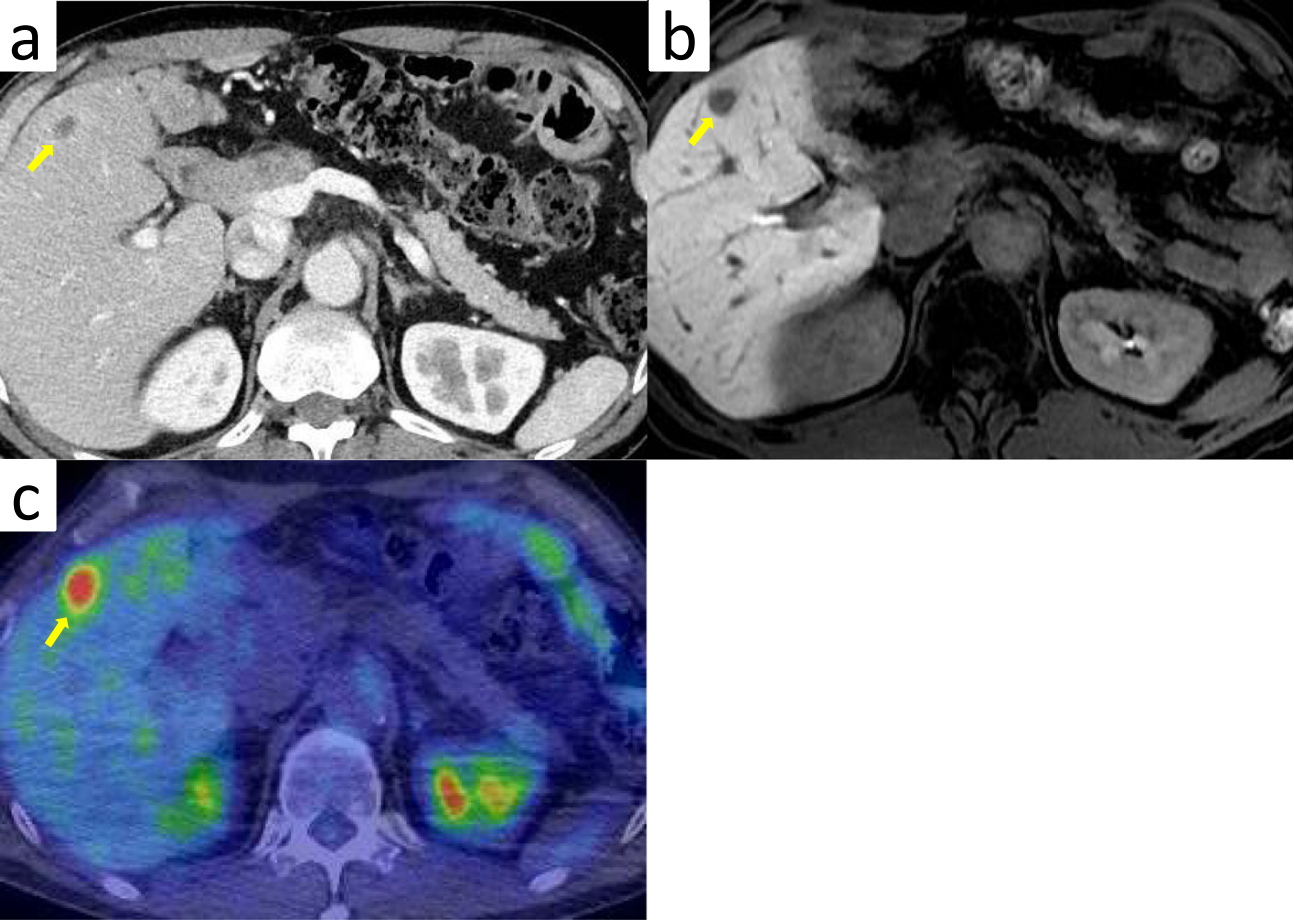
**a** Contrast-enhanced CT showing a 10-mm low-density area in liver segment 5 (arrow). **b** Gd-EOB-DPTA–enhanced MRI showing a well-defined mass in liver segment 5 (arrow). **c** PET/CT showing the liver mass with high fluorine-18-fluorodeoxyglucose uptake (arrow). CT, computed tomography; Gd-EOB-DPTA, gadolinium-ethoxybenzyl-diethylenetriamine; MRI, magnetic resonance imaging; PET, positron emission tomography

**Fig. 5:**
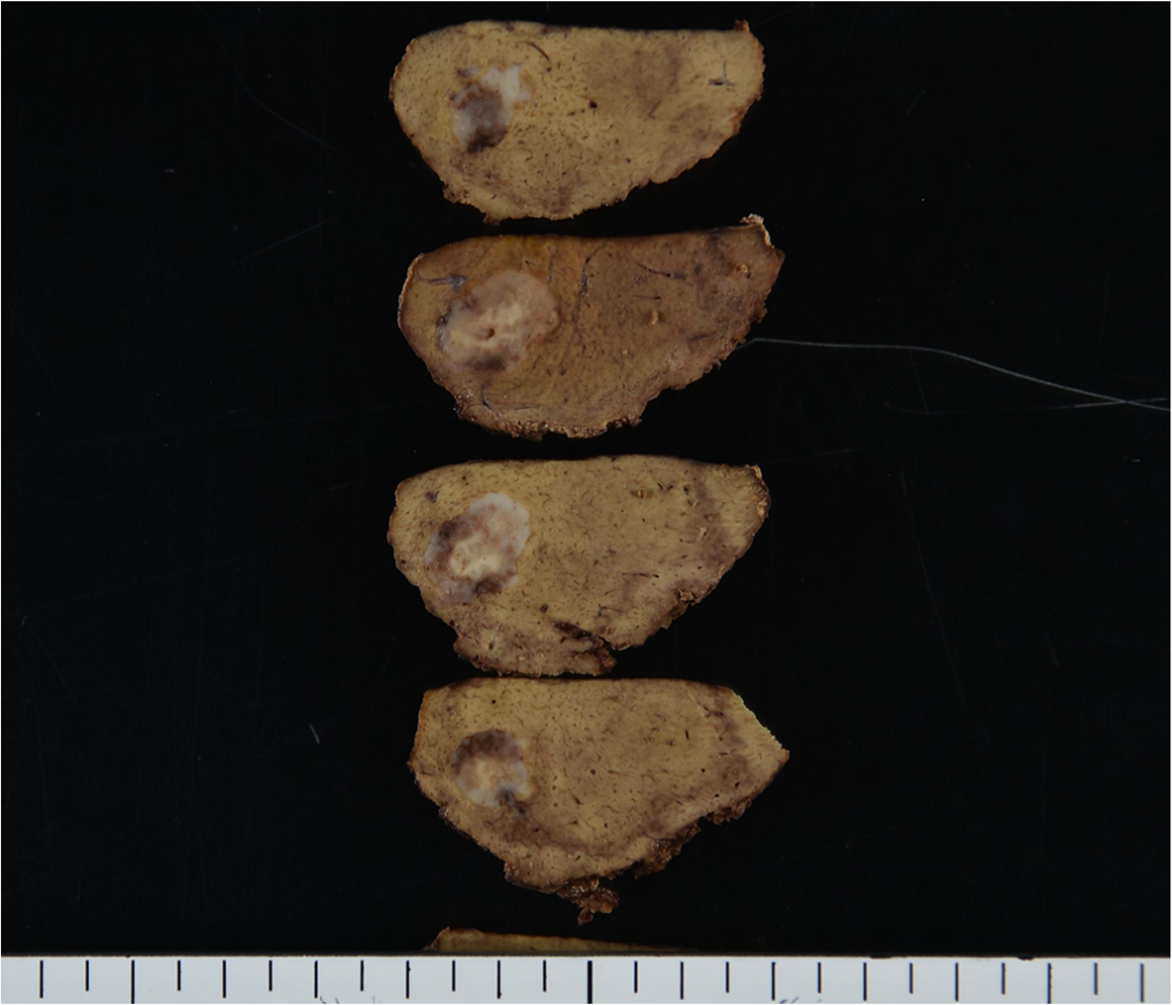
Macroscopic examination of the revealed specimen showed a nodular 10 × 9 mm tumor under the liver subcapsular region

**Fig. 6:**
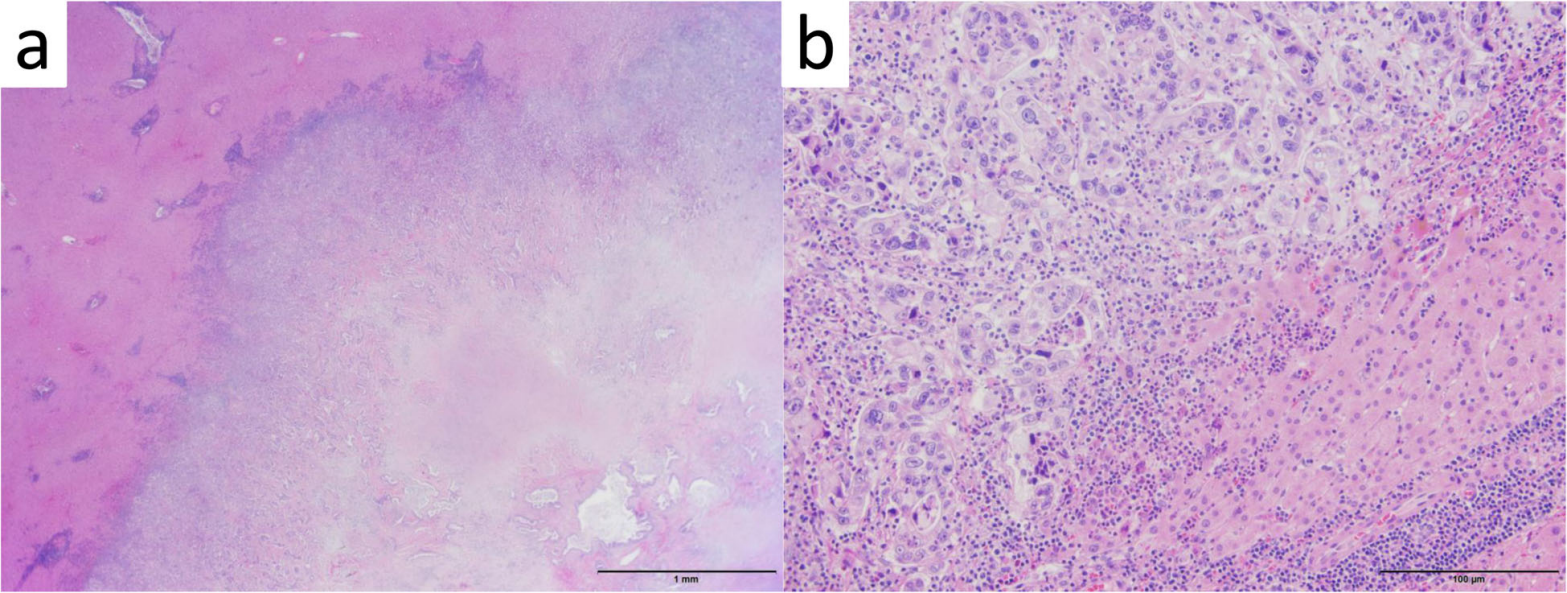
Histopathological findings (hematoxylin–eosin staining) showing a well to moderately differentiated adenocarcinoma compatible with a metastasis of pancreatic ductal adenocarcinoma. **a** × 4 and **b** × 40 original magnification

**Fig. 7:**
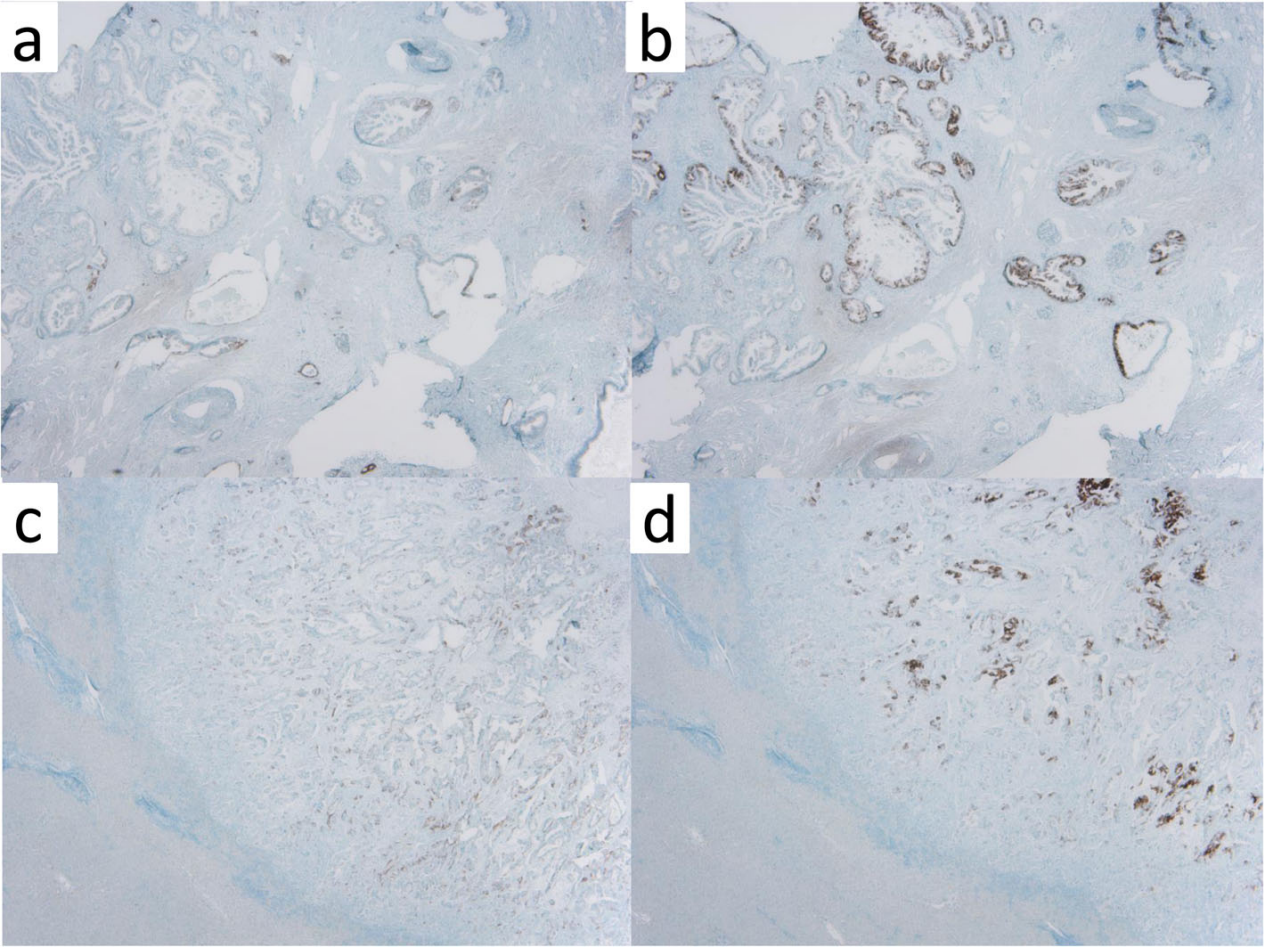
Histopathological findings (immunostaining × 4 original magnification) showing positive cytokeratin 17 and MUC5AC results in the primary lesion (**a**, **b**) and metastatic lesion (**c**, **d**)

A 51-year-old man was admitted to our hospital for further examination and treatment, including surgery for a pancreatic tumor, detected using abdominal ultrasound (US) and computed tomography (CT). He had no history of malignancy, and physical and laboratory examinations, including tumor markers, revealed no specific findings. Contrast-enhanced CT and gadolinium-ethoxybenzyl-diethylenetriamine pantaacetic acid (Gd-EOB-DPTA)–enhanced magnetic resonance imaging (MRI) revealed a nodule that was gradually contrasted in the pancreatic head (Fig. 1a–d). No enlarged lymph nodes or distant metastases were evident. Endoscopic retrograde cholangiopancreatography showed no pancreatic duct dilation or irregularity. Pancreatic juice cytology showed no malignancy. Endoscopic US revealed a well-defined hypoechoic 19 × 13 mm mass on the ventral side of the pancreatic head. Accordingly, pancreatic cancer was suspected and PD with D2 lymph node dissection was performed. A macroscopic examination of the resected specimen showed a well-circumscribed 21 × 15 mm nodule in the pancreatic head (Fig. 2). A postoperative pathological examination revealed a well-differentiated invasive ductal adenocarcinoma with lymphatic invasion and lymph node metastasis (N1; 1/19) (Fig. 3). The tumor was classified as stage IIB (pT2N1M0) according to the 8th edition of the International Union Against Cancer Tumor Node Metastasis classification. The patient experienced postoperative pancreatic fistula (Clavien–Dindo grade IIIa) [3], which was treated with drainage and antibiotic agents, and was discharged on postoperative day 50. Two months after the surgery, adjuvant chemotherapy containing gemcitabine was administered at a dose of 1700 mg (1000 mg/m^2^) on days 1, 8, and 15 every 4 weeks for 1 year. Thereafter, the carbohydrate antigen 19-9 (CA19-9) level was within the normal range and a follow-up CT revealed no local recurrence or distant metastasis. However, at 8 years after the first surgery, the serum CA19-9 level was elevated (130.3 U/mL). Additionally, CT identified a 10-mm low-density area in liver segment 5 (Fig. 4a), while Gd-EOB-DPTA–enhanced MRI revealed a well-defined mass in the area (Fig. 4b). Positron emission tomography/CT also revealed high fluorine-18-fluorodeoxyglucose uptake only in this hepatic tumor (Fig. 4c). Furthermore, upper and lower gastrointestinal endoscopy revealed no malignant findings. No other distant metastases were observed. Accordingly, he was diagnosed with liver metastasis of PDAC. Because the liver metastasis was isolated long after the initial surgery, we decided to resect it using laparoscopic partial hepatectomy of segment 5 at 8 years and 1 month after the PD. A macroscopic examination of the resected specimen revealed a 10 × 9 mm nodular tumor under the liver subcapsular region (Fig. 5). A postoperative pathological examination demonstrated well to moderately differentiated adenocarcinoma (Fig. 6a, b) with no continuity between the liver tumor and the peripheral bile duct. Additionally, immunostaining was positive for cytokeratin 17 (CK17) and MUC5AC, and the immunostaining findings of the metastatic lesion were consistent with those of the primary lesion (Fig. 7). Based on the above results, the final pathological diagnosis was liver metastasis of PDAC. We planned to administer chemotherapy for 6 months. However, the patient strongly hoped to continue adjuvant chemotherapy beyond that period. For this reason, he received adjuvant chemotherapy for 1 year. No evidence of recurrence was noted at 11 years after the PD and 3 years after the hepatic resection.

## Discussion

The prognosis of postoperative recurrence of PDAC is extremely poor [4]. A recent guideline for metastatic PDAC recommended intensive chemotherapy regimens such as FOLFIRINOX (fluorouracil, leucovorin, irinotecan, and oxaliplatin) or gemcitabine plus nanoparticle albumin-bound paclitaxel as the first-line treatment [5]. For this reason, liver metastases of PDAC are not generally indicated for surgery [6]. Some researchers insist that the resection of liver metastasis is not beneficial [7], while others suggest the use of hepatectomy in selected patients [8–10]. Because the selection criteria remain vague, patients with liver metastases of PDAC who would benefit from hepatectomy should be identified. At the time of the hepatectomy in the present case, the Japanese clinical practice guidelines for pancreatic cancer 2016 [11] had not yet stated the indication for liver resection for liver metastasis. Thus, in the present case, we selected partial hepatectomy without administering additional chemotherapy after the diagnosis of liver metastasis of PDAC because the liver metastasis was isolated and no recurrence was identified for 8 years after the radical surgery. It is difficult to discriminate intrahepatic cholangiocarcinoma (ICC) from liver metastasis of PDAC due to histological similarities between them. Lok et al. [12] reported higher frequencies of CK17 and MUC5AC in PDAC than in ICC (60% vs 12% and 67% vs 12%, respectively). In the present case, CK17 and MUC5AC immunostaining findings were positive, and the immunostaining findings of the metastatic lesion were consistent with those of the primary lesion. Additionally, there was no continuity between the liver tumor and the peripheral bile duct. Accordingly, we considered the liver tumor metastatic PDAC. A search of the literature revealed that only 6 patients survived more than 3 years after the initial surgery, hepatectomy for metachronous liver metastasis, including the present case (Table 1) [13–16]. Almost all patients exhibited a relatively long time to relapse, and these results suggest that a longer time to relapse may be associated with a longer survival time. Additionally, all patients had a solitary metastasis. Hackert et al. [9] reported a 5-year survival rate of 8.1% after surgery for liver oligometastases. Zanini et al. [17] demonstrated that patients with metachronous liver metastasis had a significantly longer median survival than that of patients with synchronous disease (11.4 months vs. 8.3 months, *p* = 0.038). Furthermore, in a retrospective analysis, overall survival was significantly prolonged in patients with resected liver metastasis of PDAC (median 14 months vs. 8 months, *p* < 0.001) [10]. These findings indicate that hepatectomy might confer long-term survival when the liver metastasis is isolated and the time to postoperative recurrence is long. In addition, Voss et al. uncovered that the resection of liver metastasis of PDAC can be safely performed [18]. Therefore, for patients with liver metastasis of PDAC, onset timing, number of lesions, and time to recurrence may be useful criteria for surgery. Further studies are needed to determine the indications for liver resection.

**Table 1:** Previous reports of the patients resected metachronous liver metastasis of PDAC and survived more than 3 years

No.	Author	Year	Age	Sex	Part	Initial Operaion	Histology	UICC stage	Number of liver metastasis	Recurrent Operation	Additional therapy after hepatectomy	Time to Recurrence (year)	Survival (year)	Status
1	Lemke J [13]	2011	48	F	unknown	unknown	unknown	IB	solitary	unknown	no	3	17	alive with relapse-free
2	IIDA [14]	2014	63	F	head	SSPPD	well	III	solitary	PH	no	1.9	6	alive with relapse-free
3	Fujisaki [15]	2015	62	M	head	PD	unknown	III	solitary	SH	AC (GEM+S-1)	0.8	3.2	dead with relapse in lymph node
4	Fujisaki [15]	2015	51	M	head	PD	unknown	unknown	solitary	PH	AC (S-1, FOLFIRINOX)+ RT	1.4	3.2	alive with relapse in lymph node
5	Omichi [16]	2015	68	F	body	DP	poorly	IIB	solitary	PH	no	1.5	5.4	alive with relapse-free
6	Present case	2020	51	M	head	PD	well	IIB	solitary	PH	AC (S-1)	8	12.1	alive with relapse-free

*PDAC* pancreatic ductal adenocarcinoma, *PD* pancreatoduodenectomy, *SSPPD* stomach-preserving pancreatoduodenectomy, *DP* distal pancreatectomy, *SH* segment heptectomy, *PH* partial hepatecomy, *Lap* laparoscopic, *AC* adjuvant chemotherapy, *RT* radiotherapy

PDAC involves the early capability of cells to migrate to distant organs, and many patients have metastatic disease at the time of PDAC diagnosis [19]. For this reason, controlling micrometastases using systemic chemotherapy is important for patient long-term survival. The literature search [13–16] (Table 1) revealed that adjuvant chemotherapy has been used in many cases and that multidisciplinary therapy including chemotherapy may contribute to long-term survival. In PDAC, it is possible for other metastases to appear shortly after the diagnosis, even if none are evident at the time of diagnosis. Therefore, it may be important to determine the effectiveness of hepatectomy after chemotherapy and follow patients for a certain duration.

## Conclusions

Here, we reported an extremely rare case of metachronous liver metastasis of PDAC after radical surgery in a patient who survived without a recurrence for 11 years after PD and 3 years after hepatectomy. The present findings suggested that hepatectomy may confer long-term survival in selected patients. The time to postoperative recurrence and the number of liver metastases may be useful criteria for deciding whether to perform hepatic resection.

## Data Availability

Not applicable.

## Abbreviations

CA19-9: Carbohydrate antigen 19-9
CT: Computed tomography
Gd-EOB-DPTA: Gadolinium-ethoxybenzyl-diethylenetriamine pantaacetic acid
ICC: Intrahepatic cholangiocarcinoma
MRI: Magnetic resonance imaging
PD: Pancreatoduodenectomy
PDAC: Pancreatic ductal adenocarcinoma
US: Ultrasound
