# Correction to: Long-term survival after hepatectomy for metachronous liver metastasis of pancreatic ductal adenocarcinoma: a case report

**DOI:** 10.1186/s40792-020-01070-x

**Published:** 2020-12-07

**Authors:** Chikanori Tsutsumi, Toshiya Abe, Tomohiko Shinkawa, Kazuyoshi Nishihara, Sadafumi Tamiya, Toru Nakano

**Affiliations:** 1 Department of Surgery, Kitakyushu Municipal Medical Center, 2-1-1 Bashaku, Kokurakita-Ku, Kitakyushu, 802-0077 Japan; 2 Department of Pathology, Kitakyushu Municipal Medical Center, Kitakyushu, Japan

## Correction to: Surg Case Rep (2020) 6:157 https://doi.org/10.1186/s40792-020-00924-8

Following the publication of the original article [1], an error was identified in the “Case presentation” section. The patient’s age in the first sentence is currently 69-year-old, however, it should be 51-year-old.

The original article has been corrected.

## References

[1] Tsutsumi C, Abe T, Shinkawa T, Nishihara K, Tamiya S, Nakano T (2020). Long-term survival after hepatectomy for metachronous liver metastasis of pancreatic ductal adenocarcinoma: a case report. Surgical Case Reports.

